# 3D Rotational Angiography in Follow-Up of Clipped Intracranial Aneurysms

**DOI:** 10.1155/2014/935280

**Published:** 2014-01-20

**Authors:** Subhash Kumar, Shailesh B. Gaikwad, Nalini Kant Mishra

**Affiliations:** ^1^Department of Radiodiagnosis, All India Institute of Medical Sciences, Phulwari Sharif, Patna, Bihar 801505, India; ^2^Department of Neuroradiology, All India Institute of Medical Sciences, Ansari Nagar, New Delhi 110029, India

## Abstract

*Introduction*. 3D Rotational Angiography (RA) is indispensable for evaluation of intracranial aneurysms, providing infinite viewing angles and defining the aneurysm morphology. Its role in follow-up of clipped aneurysms remains unclear. We aimed to compare the aneurysm residue/recurrence detection rate of 3D RA with 2D digital subtraction angiography (DSA). *Methods*. 47 patients harboring 54 clipped aneurysms underwent both 2D DSA and 3D RA. The residual/recurrent aneurysms were classified into five grades and the images of both modalities were compared. *Results*. The residual/recurrent aneurysm detection rate was 53.70% (29/54 aneurysms) with 2D DSA and 66.67% (36/54 aneurysms) with 3D RA (*P* = 0.05). In 12 aneurysms, 3D RA upgraded the residue/recurrence among which nine had been completely not detected on 2D DSA and were found to have grade one or two residual necks on the 3D RA, and, in three cases, a small neck on 2D DSA turned out to be aneurysm sac on 3D RA. In a total of 5 aneurysms, the classification was downgraded by 3D RA. *Conclusion*. 3D RA picks up more aneurysm residue/recurrence; hence, both 2D DSA and 3D RA should be performed in follow-up evaluation of clipped aneurysms.

## 1. Introduction

X-ray digital subtraction angiography (DSA) has been the gold standard investigation in the evaluation of intracranial aneurysms both before and after surgical clipping [1–3]. Two-dimensional (2D) DSA has been used over the years and has been supplemented by three-dimensional (3D) Rotational Angiography (RA) which has been shown to be superior in evaluation of untreated aneurysms with regard to aneurysm detection rate, morphology delineation, and size estimation, features which have a direct implication in the treatment planning. Thus, now 3D RA is being labelled as the “new gold standard” [4–9].

Considering the added advantage of 3D RA over 2D DSA in evaluation of native aneurysms, it follows that the same could also be applicable to evaluation of clipped aneurysms. We undertook the study to assess whether 3D RA compared favourably to 2D DSA in picking up aneurysm remnant/recurrence.

## 2. Materials and Methods

47 consecutive patients referred to the authors for follow up angiograms after surgical clipping were taken into study and underwent both 2D DSA and 3D RA on a Neurostar Biplane Neuroangiography suite (Siemens, Erlangen, Germany). The 2D views taken were anteroposterior (AP), lateral (Lat), and right and left anterior obliques (RAO, LAO) and a view with specific angle determined by preoperative DSA showing the neck of the aneurysm. 3D RA was subsequently performed, acquired data transferred to the Work Station (InSpace, Leonardo VB30B, Siemens AG, Berlin and Munchen, 2003) automatically, and reconstruction process started immediately.

3D Reconstruction and rendering: various rendering techniques [10–14] were employed in each case: maximum intensity projection (MIP), surface shaded display (SSD), volume rendered transparent (VRT), and two-color coils and clips.

The aneurysm residue/recurrence was assessed by two authors (SK and NKM) and classified as per Sindou et al. [15] into 5 grades: Grade 1: less than 50% of neck implantation; grade 2: more than 50% of neck implantation; grade 3: residual lobe from a multilobulated sac; grade 4: residual portion of the sac, less than 75% of aneurysmal size; grade 5: residual portion of the sac more than 75% of aneurysm's size. 

*Statistical Analysis.* Wilcoxon signed-rank test was used to compare the different grades of aneurysm residue/recurrence in 2D DSA and 3D RA.

## 3. Results

A total of 47 patients were evaluated, who underwent angiograms of 51 vessels harbouring 54 aneurysms (4 patients had two aneurysms in the same injected vessel).

The patients' age varied from 14 years to 74 years (maximum were between 45 and 65 years) and the male to female ratio was 1 : 1.85.

**Figure 1:**
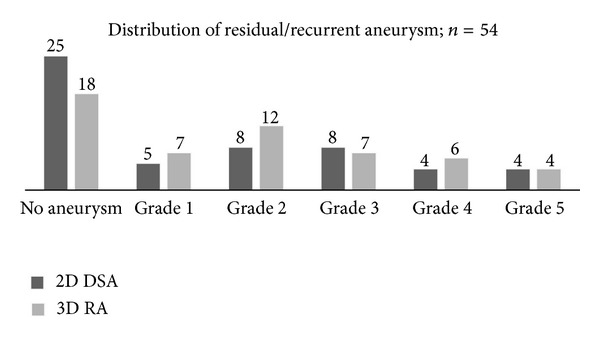
Grades of aneurysm residue/recurrence in 2D digital subtraction angiography and 3D rotational angiography.

The aneurysms' primary distribution was anterior communicating artery—20, middle cerebral artery—11, paraclinoid internal carotid artery—9, posterior communicating artery—8, distal anterior cerebral artery—3, posterior cerebral artery, anterior inferior cerebellar artery, and basilar tip—1 each.  Figure 1 shows the presence/absence and the different grades of residual/recurrent aneurysm in a head-to-head comparison of 2D DSA and 3D RA. The residual/recurrent aneurysm detection rate was 53.70% (29/54 aneurysms) with 2D DSA and 66.67% (36/54 aneurysms) with 3D RA. The results of the Wilcoxon signed rank test were *W* = 83, *n* = 17, *z* = 1.95, area between ±*z* = 0.9488, *P*(1-tail) = 0.0256, and *P*(2-tail) = 0.0512 (just about significant). In 12 aneurysms, 3D RA upgraded the residue/recurrence, among which nine had been completely not detected on 2D DSA and were found to have grade one or two residual necks on the 3D RA, and, in three aneurysms, a small neck on 2D DSA turned out to be aneurysm sac on 3D RA.

In a total of 5 aneurysms, the classification was downgraded by 3D RA, one each from grade 1 to 0, grade 2 to 0, grade 3 to 1, grade 3 to 2, and grade 4 to 3.

## 4. Discussion

By simultaneously rotating the X-ray tube and the image intensifier during the intra-arterial injection of contrast media, after having taken a spin of mask images, RA and 3D reconstructed images enable us to obtain accurate information regarding the cerebral arteries and the pathologies they possess by visualizing them from different perspectives and avoiding vessel overlap.

The evaluation of the result of aneurysm clip placement is important because the complete exclusion of aneurysms from the circulation is the aim of the operation. The incidence of recurrent aneurysm after successful clip placement has been reported to be 1.5–2.9% [16, 17], while the reported incidences of residual aneurysms are between 4% and 8% [18–21] and the risk of bleeding from residual aneurysm is believed to be 1-2% [16, 18].

2D DSA has been used as the standard for the follow-up imaging of clipped aneurysms. However, the small remnant/recurrent aneurysms are often difficult to detect and evaluate because of the clip mass and overlap of adjacent vessels.

Kang et al. [3] studied 71 patients with 88 clipped aneurysms and found 37 residual aneurysms (42%) with 3D RA as opposed to only 18 with 2D DSA. They attributed this to the excellent image quality and the various viewing angles provided by 3D angiography coupled with the ability to avoid and if necessary delete the overlapping vessels.

Murakamia et al. [22] have also published a paper wherein they assessed 67 treated (both coiled and clipped) aneurysms and found good results with regard to remnant detection rate. However, they have neither published any details nor any statistics in their paper.

We had a very high residual/recurrent aneurysm detection rate (53.70% and 66.67% with 2D DSA and 3D RA, resp.). This high percentage was possibly because many of the cases were referred to us and the operating neurosurgeon might have had a suspicion of a residue. However, we do not have correlation with intraoperative findings available for all the patients.

**Figure 2:**
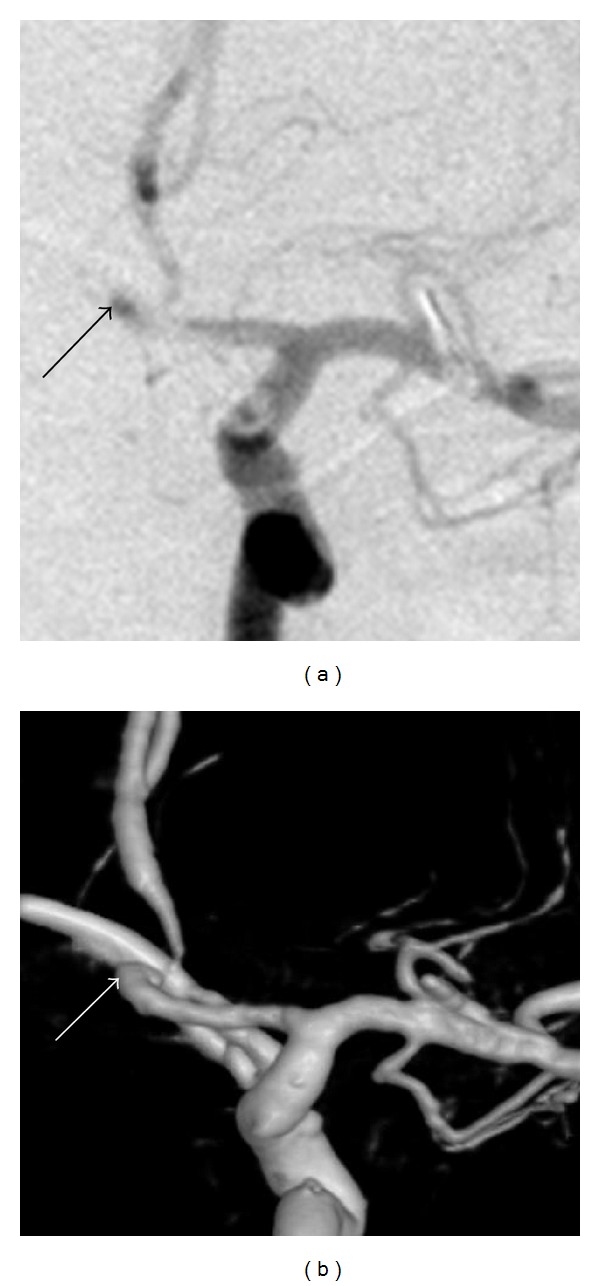
Images showing downgrading of aneurysm residue by 3D RA by showing the excellent relationship between the artery and the clip: (a) 2D DSA image showing a small remnant (black arrow), (b) 3D RA, SSD image, showing the “remnant” to be a loop of the vessel rather than aneurysm neck.

**Figure 3:**
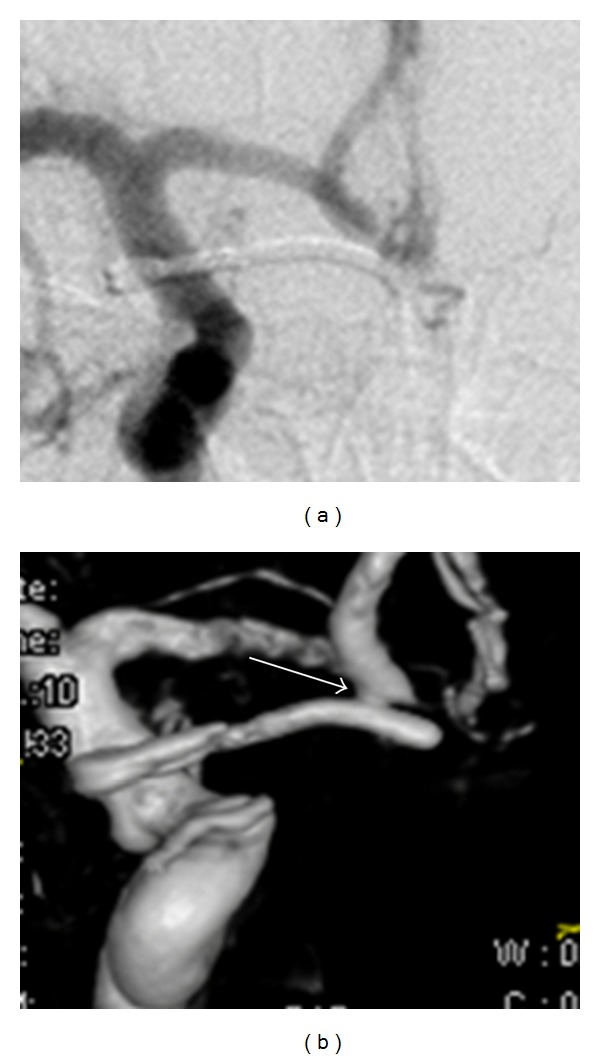
2D digital subtraction angiography (a) and surface shaded display (b) images showing a small neck in the latter while the 2D image is normal.

3D RA excellently showed the clip-artery, clip-aneurysm, and artery-aneurysm relationship (Figure 2). Small aneurysmal remnants were depicted well (Figure 3). In fact, most extra residues picked were type 1 or type 2 residual necks. Out of the 12 extra aneurysms picked up, 9 were advised a prolonged vigil for aneurysm regrowth and only 3 were advised retreatment (which the patients promptly refused).

**Figure 4:**
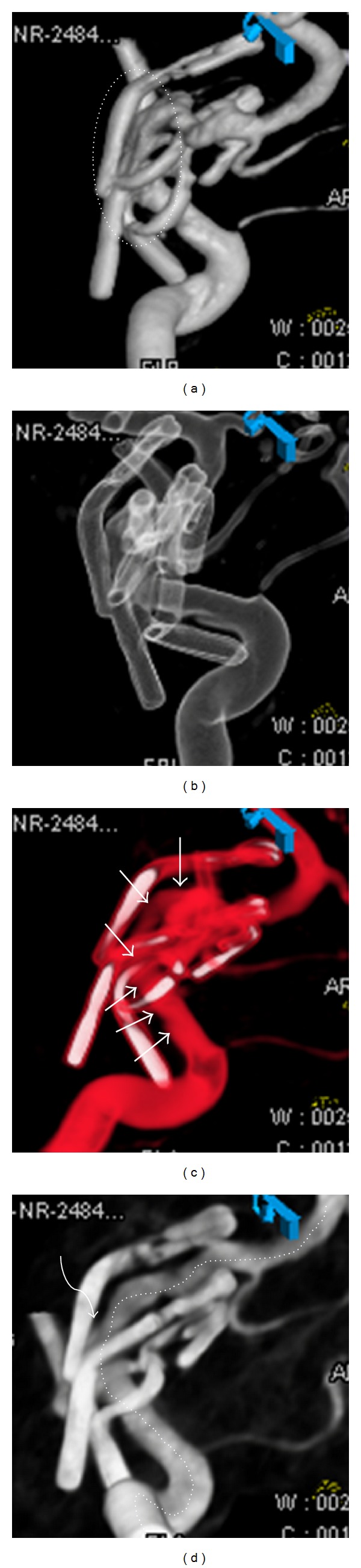
Varying visibility of the artery, aneurysm, and clips in different reconstruction techniques: (a) surface shaded display—clear visualization of the artery and multiple clips is difficult (dotted oval) and any chance of picking a small aneurysm residue is less likely, (b) transparent volume rendering—almost similar appearance, although the parent artery is visualised partially in the region of the clips, (c) two-color coils and clips—there is good visualisation of the parent artery (multiple small arrows) and the clips can be made out separately, (d) maximum intensity projection—best differentiation and visualization offered in this particular case; the entire length of the artery is well visualized (dotted freehand line over the artery), all the clips are clearly seen, and a small aneurysm neck is visualised (curved arrow).

In our study, we studied SSD, VRT, MIP and two-color, coils and clips reconstructed images in all the cases in multiple viewing angles and varied thresholds. As per our day-to-day experience, all of these commonly used techniques, namely, SSD, VRT, MIP, and two-color coils and clips have to be viewed for adequate assessment. An example (Figure 4) illustrates this fact, wherein two-color and MIP could show the artery and clip relationship well, while SSD could not, and VRT could do so only in limited manner.

Hence, a difference between these various techniques was not evaluated as all of them were found to be complementary to each other and indispensable. The final opinion formed after analyzing all the images was compared with that of 2D DSA findings.

## 5. Conclusion

3D RA picks up more residual/recurrent aneurysms than 2D DSA and both 2D DSA and 3D RA should be performed in follow-up of clipped aneurysms.
